# An Accurate Bioimpedance Measurement System for Blood Pressure Monitoring

**DOI:** 10.3390/s18072095

**Published:** 2018-06-29

**Authors:** Toan Huu Huynh, Roozbeh Jafari, Wan-Young Chung

**Affiliations:** 1 Department of Electronic Engineering, Pukyong National University, Busan 48513, Korea; tanhinhsg@gmail.com; 2 Departments of Biomedical Engineering, Computer Science and Engineering, and Electrical and Computer Engineering, Texas A&M University, College Station, TX 77843, USA; rjafari@tamu.edu

**Keywords:** blood pressure, bioimpedance, pulse wave velocity, pulse transit time, wearable structure

## Abstract

One potential method to estimate noninvasive cuffless blood pressure (BP) is through measurement of pulse wave velocity (PWV), which can be characterized by measuring the distance and the transit time of the pulse between two arterial sites. To obtain the pulse waveform, bioimpedance (BI) measurement is a promising approach because it continuously reflects the change in BP through the change in the arterial cross-sectional area. Several studies have investigated BI channels in a vertical direction with electrodes located along the wrist and the finger to calculate PWV and convert to BP; however, the measurement systems were relatively large in size. In order to reduce the total device size for use in a PWV-based BP smartwatch, this study proposes and examines a horizontal BI structure. The BI device is also designed to apply in a very small body area. Our proposed structure is based on two sets of four-electrode BI interface attached around the wrist. The effectiveness of our system and approach is evaluated on 15 human subjects; the PWV values are obtained with various distances between two BI channels to assess the efficacy. The results show that our BI system can monitor pulse rate efficiently in only a 0.5 × 1.75 cm^2^ area of the body. The correlation of pulse rate from the proposed design against the reference is 0.98 ± 0.07 (*p* < 0.001). Our structure yields higher detection ratios for PWV measurements of 99.0 ± 2.2%, 99.0 ± 2.1%, and 94.8 ± 3.7% at 1, 2, and 3 cm between two BI channels, respectively. The measured PWVs correlate well with the BP standard device at 0.81 ± 0.08 and 0.84 ± 0.07 with low root-mean-squared-errors at 7.47 ± 2.15 mmHg and 5.17 ± 1.81 mmHg for SBP and DBP, respectively. Our results inform future designs of smart watches capable of measuring blood pressure.

## 1. Introduction

**Figure 1:**
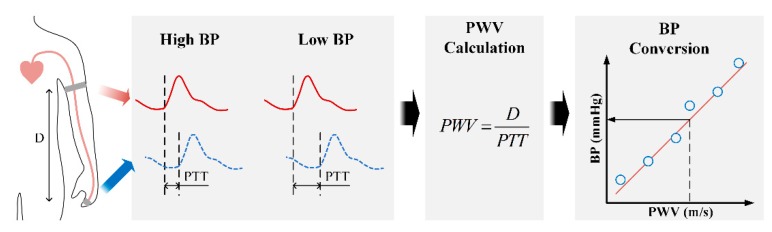
Methodology of pulse wave velocity (PWV)-based blood pressure (BP) estimation.

High blood pressure (BP) is a principal indicator of several cardiovascular disorders including hypertension. BP monitoring and management are essential to provide more effective care to patients and avoid large financial tolls and reduce the fatality of cardiovascular disorders. There are some existing noninvasive BP measurement devices that can be used at home; however, they require a cuff or they do not measure BP continuously. In order to develop a wearable device that can measure BP continuously, pulse wave velocity (PWV) and pulse transit time (PTT) have been investigated [1]. PWV is the velocity of the pulse traveling between two arterial sites. By measuring the pulse waveforms at two different sites, PWV values can be obtained using the ratio of the known distance and the pulse transit time (PTT) between two locations. Increasing BP level increases the PWV and decreases PTT values. As shown in Figure 1, on the basis of that relationship and under some assumptions, PWV and PTT can be utilized to estimate BP [2].

A common way to measure PWV is using the electrocardiography (ECG) method at the heart and photoplethysmography (PPG) method at the finger or the toe. However, this system is bulky and inconvenient for patients [3]. To realize compact devices, systems located at the wrist and the finger have been studied. There are several main pulse waveform measurements that can be applied at the wrist, such as accelerometers, pressure sensors, PPG, and bioimpedance (BI) [4,5,6]. Accelerometers cannot measure static accelerations occurring at the low frequencies commonly found in human motion. Pressure sensors are too sensitive to temperature variation and vibration. In case of PPG sensors, the need of a light-emitting source and a photodetector is a disadvantage in that it sometimes consumes more power and cannot be fabricated into an integrated circuit (IC). Another challenge is the requirement on ensuring that the PPG sensors must remain in good contact with the skin.

In order to monitor the pulse waveform, BI measurement is a promising method. By injecting a very small electric current to the human skin, the BI method can monitor the change in skin impedance by voltage measurement using two pairs of electrodes. BI requires significantly less power compared to PPG sensors for the same level of accuracy [7]. In addition, a change in BP can be characterized by the change in the arterial cross-sectional area, which is monitored by the change in arterial impedance as follows:

(1) $$\Delta A\approx -\rho_{b}L\Delta Z/Z_{b}^{2}$$

where ∆*A* is the change in the arterial cross-sectional area, ∆*Z* is the change in measured impedance, *ρ_b_* is the resistivity of blood, and *Z_b_* represents the basal impedance of the segment. Thus, BI measurement can capture certain notable aspects for BP variations.

**Figure 2:**
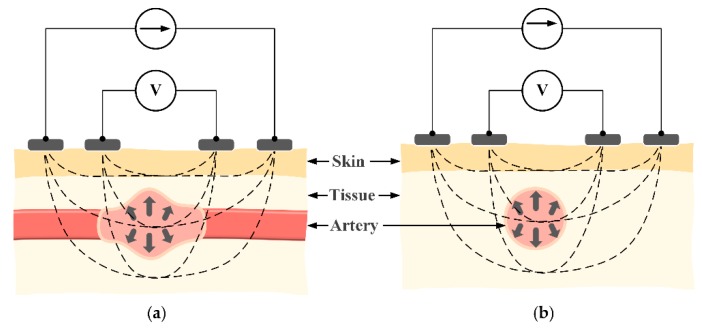
Principle of bioimpedance (BI) measurement with alternating current flowing through external electrodes in (**a**) a vertical structure and (**b**) a horizontal structure.

**Figure 3:**
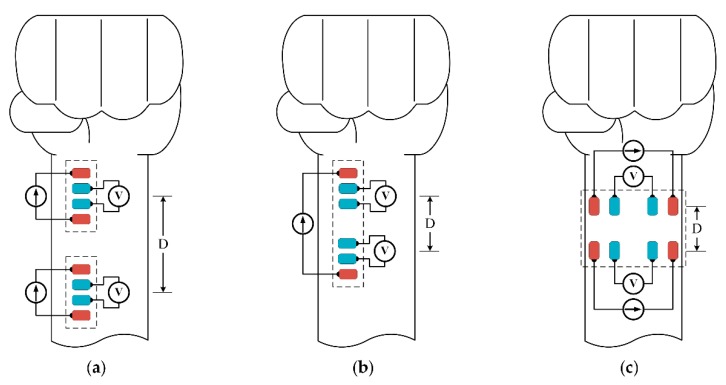
Distribution of electrode sites on the forearm for PWV measurement with (**a**) long distance; (**b**) short distance in the vertical direction; and (**c**) short distance in the horizontal direction.

To measure the impedance waveform at the wrist, four electrodes are placed above the radial artery in a vertical direction (Figure 2). This structure has shown a strong measured signal because most of the intensity of the electric current passes through the biological tissue. However, the vertical structure will need to be relatively large in size [8,9]. This issue can be mitigated by switching to a horizontal configuration. As shown in Figure 3c, with the same distance between two BI channels, the horizontal structure shows an advantage in length compared to the vertical structure, which is suitable for a smartwatch form factor. However, because the electric current does not fully pass through the biological tissue, the horizontal structure may yield a weak signal in some areas. With these considerations in mind, this paper proposes to employ the horizontal structure at the wrist to calculate PWV values and compare those values to BP levels. In order to evaluate the feasibility of the proposed approach, the horizontal BI structure was placed in different body areas at the wrist. To assess the feasibility, some analytical parameters were measured and compared to standard approaches.

## 2. Materials and Methods

### 2.1. Hardware Design

**Figure 4:**
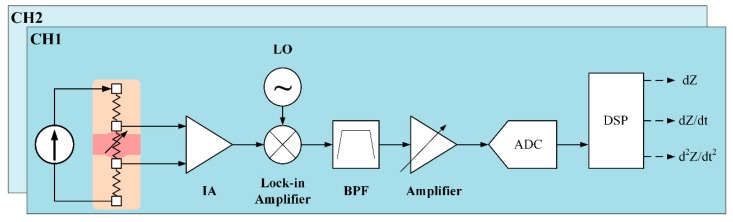
The designed system for two BI measurements in a small body area.

Our proposed system for two BI channels is shown in Figure 4. For a constant current source, a Wien-bridge oscillator is utilized to generate sine waves of 500 μA at 100 kHz. This very small current is safe for the human body, and is within the safety guidelines, and is generated at high frequency to deeply propagate into the skin. The oscillator is based on a two-stage RC coupled amplifier circuit. A connection between the inverting input terminal and the output of the amplifier allows adjusting the gain via the resistors. The noninverting terminal is connected with a series and parallel circuit of resistors and capacitors. The RC Wien Bridge network is combined with the positive feedback and has zero phase shift at the intended frequency. Therefore, at the selected resonant frequency, the voltages in both terminals will be equal. Their voltages are in-phase so that the positive feedback will remove the negative feedback. As a result, the output of the operational amplifier generates an oscillation waveform [10]. To detect a very small change in arterial impedance, an ultra-low noise instrumentation amplifier (IA) (AD8429, Analog Devices, Norwood, MA, USA) is used. This IA performs sufficiently well in measuring tiny signals with low input noise performance of 1 nV/$\sqrt{\mathrm{Hz}}$ and high common-mode rejection ratio. To amplify, a 40-dB gain is required. To achieve wide input common-mode range and low power consumption, capacitive-coupled topology is applied at the IA input pins. After obtaining the small signal, the next stage is a lock-in amplifier (AD630, Analog Devices, USA) to separate a small, narrow-band impedance variation from the carrier signal at 100 kHz and interfering noise.

In order to obtain a clear signal, an active third-order Chebyshev Sallen–Key bandpass filter is applied with cutoff frequencies at 0.1 Hz and 3 Hz. Finally, the received waveform is amplified as an input to an analog-to-digital converter module for signal processing. The sampling rate is selected at 50 kHz to detect at a very small transit time between two BI channels at an extremely short distance. For further analysis, the first and second derivatives of impedance variation waveform are obtained using differential amplifiers. The second BI channel utilizes the same design. 

The total radial impedance *Z* is the sum of the basal impedance and the impedance variation. To calculate the actual base impedance *Z_b_*, the voltage drop over the known resistor *R* of 100 Ohm is measured as an input instead of the skin impedance. To calculate the actual impedance variation *dZ*, a small sine-wave *V_S_* of 10 mV at 1 Hz is generated to use as an input of band-pass filter. The gain of the amplifier chain is obtained. Finally, with constants *K_a_* and *K_b_*, the actual radial impedance can be computed as follows:

(2) $$Z=Z_{b}+dZ=R\left(K_{a}+V_{S}\cdot K_{b}\right)$$

### 2.2. Electrode Structures

**Figure 5:**
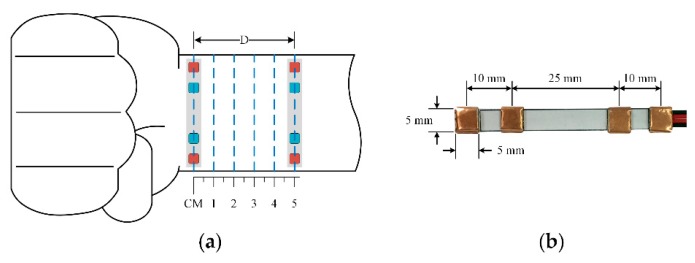
(**a**) Various locations of the BI channel placed at the wrist for the validation; (**b**) the electrode dimensions applied in this study.

A prototype horizontal structure for one BI channel is shown in Figure 5b. To demonstrate the potential for long-term monitoring, dry electrodes are employed using 3M™ conductive copper foil. At high frequency, due to the capacitor’s effects, the electrode equivalent circuit consists of the impedance associated with the electrode-skin interface *R_d_* and polarization *C_d_* in parallel. In particular, at 100 kHz, those values are 1.3 MΩ and 12 nF, respectively. Thus, the electrode-skin impedance is around 133 Ω at |*R_d_*//*C_d_*| @ 100 kHz [11]. The electrode size is 5 × 5 mm^2^. In order to improve the contact between the electrode and human skin, the thickness is determined to be 1 mm. The distances between the two voltage sensing electrodes and two current sensing electrodes are 25 mm and 45 mm, respectively. The distances between each pair of sensing electrodes are not the same because the arteries at the wrist are not in the center. The electrode dimensions are optimized to fit on the wrist size of all subjects so that the measurement site can produce good signals with the electrode location still near the arterial distribution area.

Those dimensions are designed to suit a normal wrist size and a smartwatch. The electrodes are fixed on thin general silicone plastic. For a full structure, another channel is used. The distance between the two channels can be modified. The proposed structure is placed directly on the wrist using a flexible belt to allow for stretchability.

### 2.3. Testing Protocols

To assess the feasibility of the proposed system and approach, 15 human subjects without any history of cardiovascular disease were enrolled (age: 30 ± 5 years; gender: 9 males, 6 females; height: 165 ± 10 cm; weight: 60 ± 10 kg). The study includes three main experiments. First, only one BI channel was tested and validated with a continuous reference pulse sensor. Next, two BI channels were used to determine the feasibility of PWV measurement at various distances. Finally, the distance between two BI channels was optimized and a prototype PWV-based device was designed and evaluated in comparison to a reference BP measurement device. The following tests were then conducted.

#### 2.3.1. Performance Comparison to Commercial Pulse Sensor

A BI channel was placed at various distances of 1 cm to 5 cm to the bracelet line as described in Figure 5a, while a reflected optical sensor RP520 (Laxtha, Daejeon, Korea) was placed at the index finger as a reference. All ipsilateral measurements were performed with the subject in a seated position with the arm resting on a table. For each subject, both BI and PPG waveforms were recorded over 20 s at various distances to provide five pairs of simultaneous pulse waveform measurements. To evaluate the performance of the proposed BI system, the correlation coefficients (r) and the signal-to-noise ratio (SNR) were calculated in comparison to the signals acquired from the reference device. In addition, the ensemble impedance waveforms were displayed at different locations on the forearm, and the impedance variations were computed.

#### 2.3.2. Validation of PWV Measurement

To calculate PWV values, one BI channel was placed at the 0-cm line and another channel was placed and moved sequentially at distances from 1 cm to 5 cm. Thus, for each subject, five PWV values were obtained at the different distances between two channels. The waveforms were recorded over 20 s. Next, the PTT values were calculated, and finally, with each distance, PWV was computed.

Ideally, the PTT values are always positive. However, an erroneous transient delay time may still occur during measurement. Negative PTT values can be obtained because factors as the effects of motion artifacts, unstable attachment between electrodes and skin or BI capturing the pulse arrival time not on the main arteries. 

**Figure 6:**
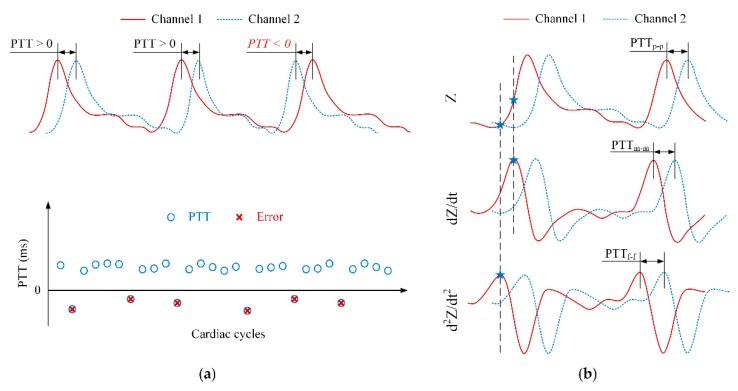
(**a**) Mistaken pulse transit time (PTT) values during the measurement process; (**b**) three types of PTT detections: peak-to-peak PTT (PTT_p-p_), middle-to-middle PTT (PTT_m-m_), and foot-to-foot PTT (PTT_f-f_).

In this study, three types of PTT detection algorithms were employed to determine the best candidate for PWV calculation with the fewest errors. As shown in Figure 6b, the peak, middle, and foot points of the impedance waveform were detected. The time intervals between peak-to-peak, middle-to-middle, and foot-to-foot of two waveforms were calculated to provide PTT_p-p_, PTT_m-m_, and PTT_f-f_ values, respectively.

To quantify the quality of the PTT detection extracted from the individual beats of the BI waveforms at various distances, a detection ratio (DR) was calculated. For a full recording, DR is defined as the ratio between the number of positive PTT values and the total number of PTT values. In other words, DR will be 100% if the recording has no negative PTT values. However, DR does not reflect the magnitude of the measured PTT or PWV values. With *N* is the total number of beats, DR can be obtained by

(3) $$\mathrm{DR}=\frac{1}{N}\sum_{i=1}^{N}{}^{1}\{{\mathrm{PTT}}_{i}\geq 0\}$$

To process the waveform, a band-pass filter (*f_pass_* = 0.5–10 Hz) was applied to smoothen the signal and reduce the noise. The low cutoff frequency was selected to eliminate the undesirable signals due to the motion artifacts, whereas the high cutoff frequency was selected to eliminate high-frequency noise and interference. Next, first-order and second-order differentiators were applied to generate the derivative BI waveforms. An automatic beat detection was then performed to detect the three points of the BI waveform: the peak, middle, and foot points. Finally, three types of PTT detections were computed. The average PTT with and without negative values over 20 s was used to calculate PWV for further analysis.

#### 2.3.3. Validation of BP Estimation

A reference BP monitoring system Oscar 2 (SunTech Medical, Morrisville, NC, USA) was used as a reference. The proposed device was placed on the wrist while the reference device was placed on the upper arm. All measurements were performed with the subject in a seated position with both devices held over the chest area to create the best comparison scenario and to prevent errors from the change due to hydrostatic pressure. Both systolic BP (SBP) and diastolic BP (DBP) from the BP reference device were recorded, while the average PTT without errors (negative values) over 20 s was used to calculate the PWV value. All those values were analyzed and evaluated for correlation coefficient and root-mean-squared-error (RMSE) to assess the estimated BP values from our proposed device.

To perturb the BP, a handgrip exercise was employed. The validation protocol included six sessions. First, each subject was instructed to relax for 10–15 seconds to record a baseline BP. Next, the increasing BP values were recorded after the subject performed the handgrip exercise. After that, the remaining five sets alternated between recovery periods and handgrip exercises.

## 3. Results

### 3.1. Validating the Pulse Waveform and Pulse Rate

**Figure 7:**
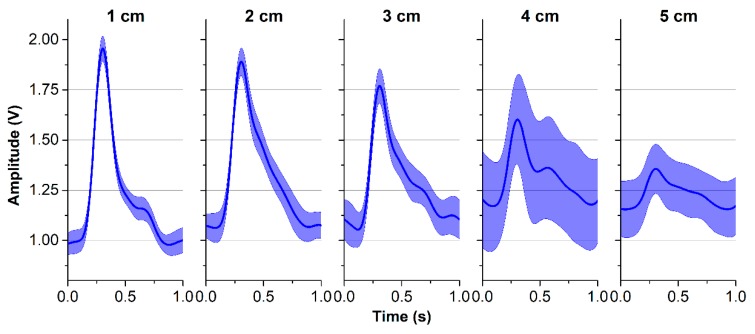
Ensemble average of BI waveforms at various locations.

Figure 7 shows the ensemble average of BI waveforms at various distances from 1 to 5 cm away from the bracelet line at the wrist. It can be seen that the BI waveform is more stable at closer distances, such as 1, 2, and 3 cm. The other areas led to BI signals with higher variations, which is manifested with a larger standard deviation (SD). Moreover, the measured waveforms in those areas were lower in amplitude in comparison to the other areas closer to the bracelet line. The actual impedance variations of all areas were computed as shown in Table 1. It is obvious that increasing distances decreased the impedance variations. At 1 cm, the measured impedance is greatest at 325.8 mΩ, whereas the minimum impedance change is 67.2 mΩ at 5 cm.

**Figure 8:**
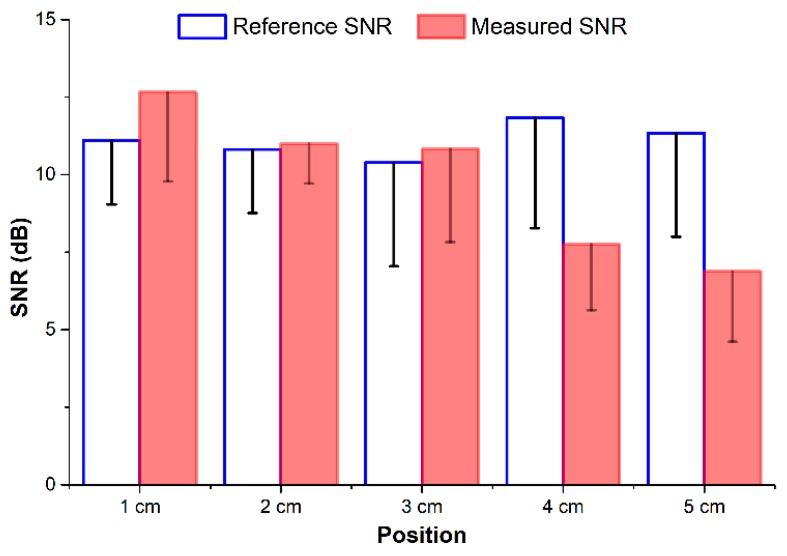
SNR values of the commercial device and the proposed BI measurements at various locations.

As shown in Figure 8, the SNRs obtained with the proposed system are nearly equal to those values from the reference device, at approximately 11 dB at 2 cm and 3 cm. The BI system achieved even higher SNR at 1 cm. However, with the areas away from the bracelet line, the BI measurements were 37% lower in SNR at approximately 7 dB at 4 cm and 5 cm.

**Figure 9:**
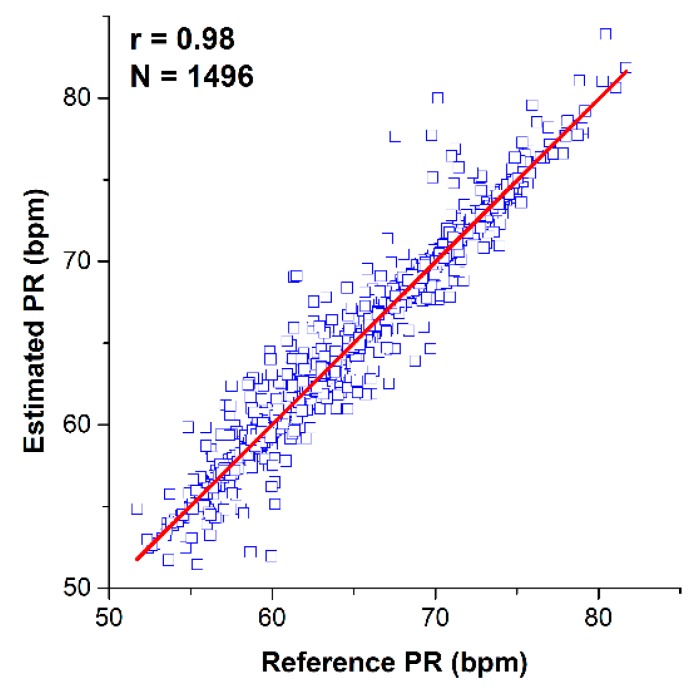
Correlation plot of estimated pulse rate (PR) from the proposed structure against the commercial device at various locations.

The group average correlation coefficients for estimating pulse rate from the proposed system against the reference device at various distances are shown in Table 1. The BI system showed strong correlations ranging from 0.82–0.98 versus the reference. The plot of estimated versus reference pulse rate values from both devices for all subjects is shown in Figure 9. A strong correlation at 0.98 for estimating pulse rate was observed.

### 3.2. Structure Optimization Results

**Figure 10:**
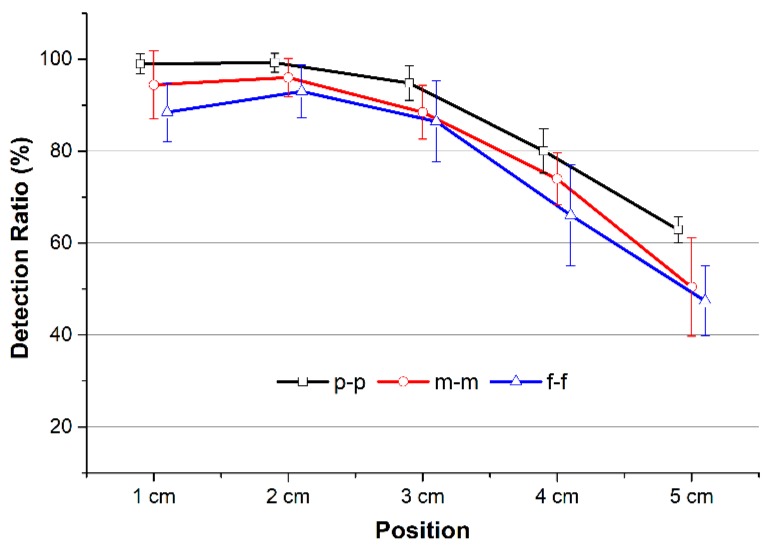
Detection ratio of three types of PTT detections at various locations.

The average DR of PTT detections at different distances between two channels can be seen in Figure 10. The DR rates dropped with increased distance. The results show that PTT_p-p_ yielded the highest DR, while PTT_f-f_ offered the lowest DR for all distances compared to the other detections. Those values were 99 ± 2.24%, 99.25 ± 2.07%, 94.78 ± 3.73%, 80.03 ± 4.84%, and 62.9 ± 2.85% for PTT_p-p_ from 1 to 5 cm, respectively. Peak-to-peak PTT detection provided 4.9%, 7.1%, and 24.7% higher in DR than middle-to-middle PTT detection; those values were 11.9%, 9.6%, and 32.6% higher compared to foot-to-foot PTT detection at 1 cm, 3 cm, and 5 cm, respectively.

**Figure 11:**
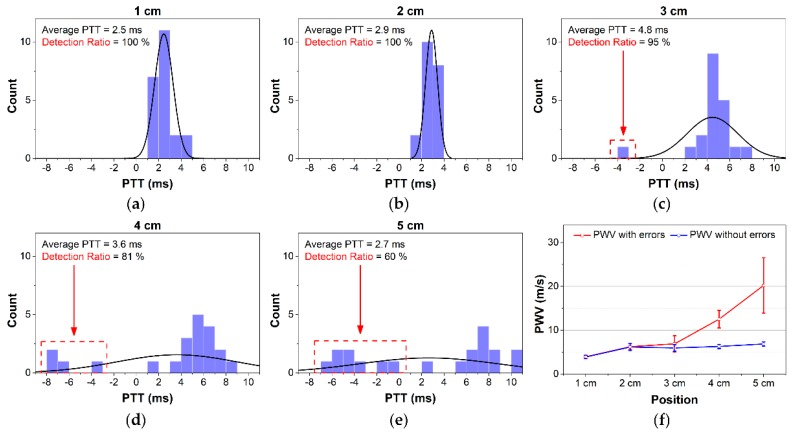
(**a**–**e**) Distribution of measured PTT at various distances over 20 s; (**f**) PWV with and without PTT errors at various distances.

Figure 11 shows histograms of measured PTT values for a subject over 20 s and the group average PWV with and without PTT errors. At 4 cm and 5 cm, it can be seen that the negative PTTs degraded the average PTT values. After eliminating the negative PTT values, the measured PWVs appeared within the normal physiological range. The average PWVs without error were 6.15 ± 0.78 m/s, 5.94 ± 0.75 m/s, 6.28 ± 0.48 m/s, and 6.85 ± 0.5 m/s from 2 cm to 5 cm, respectively. The changes in resulting PWVs were not significant at those distances. However, at 1 cm between the two channels, the PWV value was 3.87 ± 0.39 m/s, which is lower than those at other distances.

### 3.3. Correlating between the Estimated PWV and Standard BP Device

**Figure 12:**
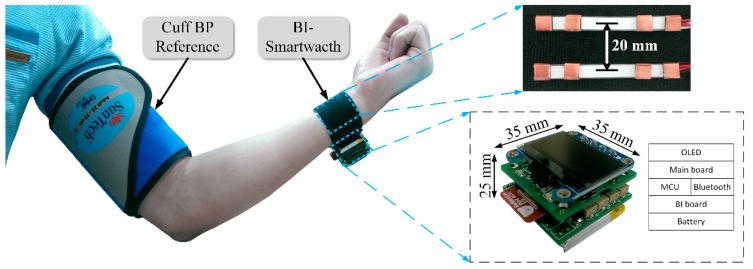
Prototype of the designed PWV-based BP smartwatch at the wrist with the BP standard device located on the upper arm.

From the results of our proposed study on estimating PWV with various BI spacing, the distance between the two channels was optimized. All distances showed good estimated PWV values except 1 cm. On the other hand, only 1 cm and 2 cm resulted in high DR and strong SNR. Therefore, the study was designed to validate the proposed structure at 2 cm between the two BI channels with the standard BP device. Figure 12 shows our prototype PWV-based BP device with a horizontal structure and BP reference monitoring. Our proposed device includes several modules including a display, controller, battery, and a wireless transceiver. The size of the design is approximately 35 × 35 × 25 mm^3^.

**Figure 13:**
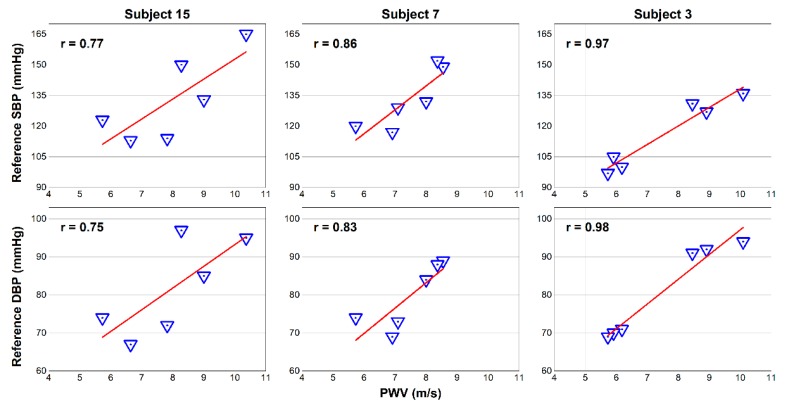
Representative correlation plots between calculated PWV and reference BP.

Table 2 shows the high correlation coefficient between PWV and both BP levels. The group average coefficients were 0.81 ± 0.08 and 0.84 ± 0.07 for SBP and DBP, respectively. Three representative subjects with different coefficients are shown in Figure 13.

After the validation with linear regression, the measured PWV values were then converted to BP levels. Compared to the reference BP device, our proposed system exhibited an RMSE of 7.47 ± 2.15 mmHg and RMSE of 5.17 ± 1.81 mmHg for SBP and DBP, respectively.

**Figure 14:**
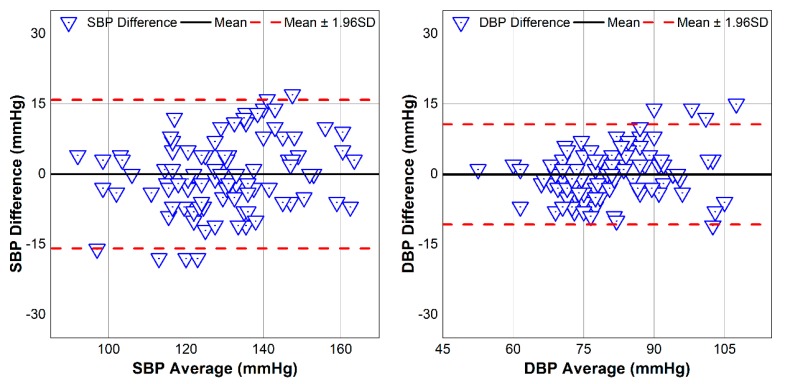
Bland–Altman plots for all subjects between estimated BP and reference BP.

The Bland–Altman plots of all predicted BP versus the reference aggregated for all subjects are shown in Figure 14. The mean is illustrated with a black solid line, and the limits of agreement (±1.96 × SD) are represented with red dashed lines. The mean ± SD of the SBP and DBP difference against the reference device are 0.01 ± 8.1 mmHg and −0.06 ± 5.46 mmHg, respectively. It is obvious that all data points from our proposed design lie within the limit of agreement. Thus, the proposed structure can estimate BP values that agree closely with those of the reference device.

## 4. Discussion

A vertical BI structure has been applied in prior studies [8,9,12] because its electric current field can cover larger arterial areas than horizontal structures. However, for PWV measurement, designs with electrodes arranged in the horizontal direction offers several advantages. First, the structure has an advantage in terms of size. It is obvious that the horizontal structure can be designed with two channels for a wearable device as the electrodes can be placed on the strap around the wrist [13]. Second, a stable BI waveform can be monitored with the hardware device as designed in this study. However, the proposed structure may not be applied to any location on the wrist. At locations 4 cm or 5 cm away from the bracelet line, the BI waveform appears less stable than at other locations. Those locations provide lower values on impedance variation, SNR, and correlation coefficient for the pulse rate compared to gold standards. The radial artery is closest to the skin surface at the areas near the bracelet line, for example, at the 1 cm location. Therefore, the quality of the BI measurements at the locations farther away from the bracelet line decreases. For this reason, many studies have applied BI measurement near the wrist to obtain more stable waveforms.

The average DR values were decreased with increasing distance. As indicated earlier, while one BI channel located at the origin of the wrist was stable, another channel yielded large errors. As a result, the calculated PTTs included more negative values at distances farther away, such as 4 cm and 5 cm. The peak-to-peak PTT provided the highest DR compared with the middle-to-middle or foot-to-foot PTT detection approaches for all distances. This result demonstrates that PTT_p-p_ is the most useful indicator for estimating PTT and PWV with our proposed system. Another indication on why the peak may be more accurate than the middle and the foot points is shown in the ensemble average BI waveforms [1].

The less-stable waveforms at 4 cm and 5 cm resulted in a larger error for PTT detection and higher PWV values. However, after removing the negative PTT values, similar PWV measurements were obtained for those compared to other positions. As described in Figure 11f, even at 1 cm, our device provides high SNR with clear waveforms, although the calculated PWVs were lower than others. This can be explained by the effects of the BI channels being too close to each other and the pulse arrival time not being detected accurately. Contact between electrodes and skin may cause a certain pressure on the artery, which is quite close to the skin surface at the 1-cm area distributing the blood flow and PWV. Thus, actual PTTs at the area near 1 cm and 0 cm were greater than normal, which result in smaller PWV [4]. This effect is negligible at the other positions. Similar results can be found in other studies [6,14]. Thus, the proposed structure at 2 cm from the wrist between two channels tracks well with both SBP and DBP.

In addition to these contributions, this study has some limitations that should be overcome in further. First, the dimension of the electrodes remained unchanged. The size of the electrode structure may affect the electric current field that passes through the artery. Additionally, the relationship between PTT, the distance of the electrodes, and the electrode-to-skin pressure should be investigated. Second, the proposed BI structure can be compared against the PPG structures to assess the advantages of each method. Third, towards realizing a complete device, the, we should take the power consumption and the total size should be optimized. Finally, the BP validation protocol should be expanded. Various BP perturbations should be applied to a larger study cohort. 

## 5. Conclusions

Our study proposed a horizontal structure for BI measurements at the wrist to optimize a BP monitoring device based on the PWV characterization. The BI hardware was designed to apply to a small body area and tested in different locations on the wrist. After comparing to a commercial pulse sensor, two BI channels were used to estimate PTT and PWV at various distances between the two channels. The wearable device was designed on the basis of the most optimal distance. In summary, our proposed design provided good tracking of BP changes in comparison to a reference device. Future work will focus on refinements to reduce the estimated BP errors. Overall, we conclude that the horizontal BI structure provides a feasible path forward for future smartwatch based BP monitoring solutions.

## Figures and Tables

**Table 1:** Impedance variation, signal-to-noise ratio (SNR) of BI measurement, and correlation coefficients of pulse rate against the commercial device under different location of forearm.

	1 cm	2 cm	3 cm	4 cm	5 cm
*dZ* (mΩ)	325.8	275.8	238.5	143.9	67.2
SNR (dB)	12.65 ± 2.87	10.99 ± 1.27	10.82 ± 2.99	7.75 ± 2.12	6.88 ± 2.27
r	0.97 ± 0.03	0.98 ± 0.02	0.93 ± 0.08	0.84 ± 0.09	0.82 ± 0.1

**Table 2:** Group average correlation coefficients and root-mean-squared-errors (RMSEs) between estimated systolic BP (SBP) and diastolic BP (DBP) from the proposed device and the standard device.

	r	RMSE (mmHg)
SBP	0.81 ± 0.08	7.47 ± 2.15
DBP	0.84 ± 0.07	5.17 ± 1.81
